# Protection Duration of COVID-19 Vaccines: Waning Effectiveness and Future Perspective

**DOI:** 10.3389/fmicb.2022.828806

**Published:** 2022-02-22

**Authors:** Chunlan Zhuang, Xiaohui Liu, Qi Chen, Yuxin Sun, Yingying Su, Shoujie Huang, Ting Wu, Ningshao Xia

**Affiliations:** ^1^State Key Laboratory of Molecular Vaccinology and Molecular Diagnostics, National Institute of Diagnostics and Vaccine Development in Infectious Diseases, Collaborative Innovation Center of Biologic Products, School of Public Health, Xiamen University, Xiamen, China; ^2^School of Life Sciences, Sun Yat-sen University, Guangzhou, China; ^3^Research Unit of Frontier Technology of Structural Vaccinology of Chinese Academy of Medical Sciences, Beijing, China

**Keywords:** waning effectiveness, protection duration, COVID-19, vaccines, SARS-CoV-2 variant

## Abstract

The coronavirus disease 2019 (COVID-19) vaccines have very successfully decreased the disease risk as we know; some key information remains unknown due to the short development history and the lack of long-term follow-up studies in vaccinated populations. One of the unanswered issues is the protection duration conferred after COVID-19 vaccination, which appears to play a pivotal role in the future impact of pathogens and is critical to inform the public health response and policy decisions. Here, we review current information on the long-term effectiveness of different COVID-19 vaccines, persistence of immunogenicity, and gaps in knowledge. Meanwhile, we also discuss the influencing factors and future study prospects on this topic.

## Introduction

An emerging infectious respiratory disease named as coronavirus disease 2019 (COVID-19) was caused by SARS-CoV-2 infection, which has spread worldwide and led to a tremendous disease burden. According to the World Health Organization (WHO), as of December 2021, there have been more than 290 million confirmed cases of COVID-19 and nearly 5.4 million deaths [World Health Organization (WHO), 2021e]. As the war against SARS-CoV-2 began, companies and research institutions have raced to develop COVID-19 vaccines at an unprecedented speed. Encouragingly, the preventive COVID-19 vaccine was developed just months after the beginning of the pandemic, which was undoubtedly a milestone in the development of human vaccines. There have been currently 23 COVID-19 vaccines approved for use in different countries [World Health Organization (WHO), 2021a], and 8 have WHO emergency use listing (EUL) authorization [World Health Organization (WHO), 2021c], including BNT162b2 (Pfizer/BioNTech, New York/Mainz, NY, United States/Germany), mRNA-1273 (Moderna Biotech, Cambridge, MA, United States), Ad26.COV2. S (Janssen, Raritan, NJ, United States), AZD1222 (Oxford/AstraZeneca, Oxford/London, United Kingdom), CoronaVac (Sinovac, Beijing, China), BBIBP-CorV (Sinopharm, Beijing, China), Covishield (Serum Institute of India Pvt. Ltd., Pune, India) and COVAXIN (Bharat Biotech, Hyderabad, India). As the widespread immunization campaign is being rapidly implemented around the world, most countries have achieved high levels of vaccination coverage with a total of more than 8,600 million doses that have been administered to date [World Health Organization (WHO), 2021e]. However, approximately 2 years since SARS-CoV-2 was first identified, there is critical information that remains unknown, let alone frequent mutations of the virus that make things more complicated. Thousands of cumulative mutations have occurred since the emergence of the virus, while only a few of them have a notable effect of the spread and virulence of the virus (Chen et al., 2020; Li et al., 2020). The WHO defined three classes of SARS-CoV-2 variants based on the risk posed to global public health, including the variant of concern (VOC), variant of interest (VOI), and variant under monitoring (VUM) [World Health Organization (WHO), 2021d]. As a class of variants that pose the greatest threat, the designated VOCs include Alpha (B.1.1.7), Beta (B.1.351), Gamma (P.1), Delta (B.1.617.2), and Omicron (B.1.1.529), according to the data updated on December 31, 2021. Although widespread mass vaccination campaigns had been implemented globally in the second half of 2021, the Delta variant that emerged in October 2020 in India led to a new wave of outbreak and became a predominance in most countries, even in Israel and Singapore where the vaccination coverages were already high (more than 50% in June 2021). In early November, 2021, the resurgent outbreak in Europe pushed the number of new COVID-19 cases past previous peaks. The spike of breakthrough infections began to raise concerns about the vaccine effectiveness (VE) against variants, but the extent to which reduced effectiveness is a result of the new variant remains not yet clear. To make matters worse, a new variant named “Omicron,” which was first reported to the WHO from South Africa on November 24, 2021, has been spreading rapidly and has ravaged South Africa and Europe before Christmas, even involved the United Kingdom and Denmark where the vaccination coverage were about 70%. All these prompted vaccinees to intend to figure out whether the immunity conferred after vaccination has waned over time. Thus, the duration of COVID-19 vaccine protection is an urgent issue that needs to be studied and explained.

## Is the Vaccine Effectiveness Really Waning Over Time?

**FIGURE 1:**
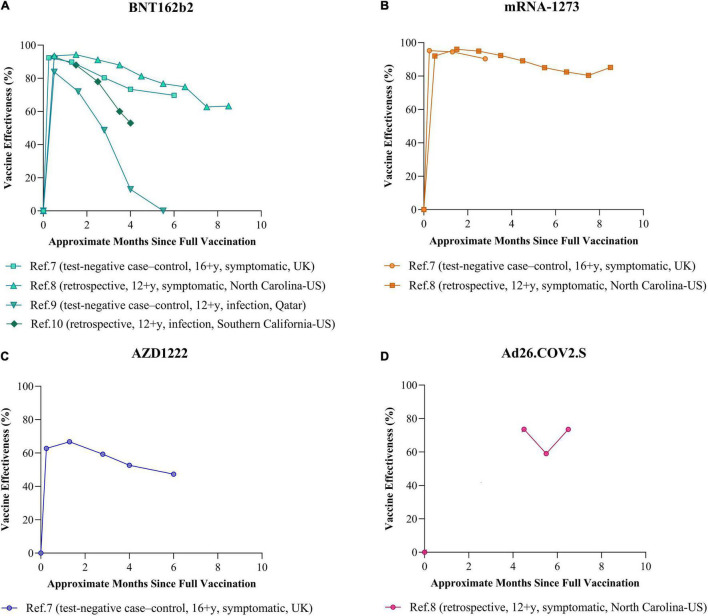
Long-term VE against infection or symptomatic disease caused by SARS-CoV-2 Delta variant. The line chart shows the trends over time of the VE against infection or symptomatic disease caused by the Delta variant after two doses of **(A)** BNT162b2, **(B)** mRNA-1273, and **(C)** AZD1222 and one dose of **(D)** Ad26.COV2.S. The reference numbers in the figure are the same as those in the text. The study as reference 7 (Andrews et al., 2021) was conducted in population aged 16 and older in the United Kingdom using test-negative case–control design. The study as reference 8 (Lin et al., 2021) was conducted in population aged 12 and older in North Carolina, United States, using a retrospective design based on the COVID-19 Surveillance System. The study as reference 9 (Chemaitelly et al., 2021) was conducted in population aged 12 and older in Qatar using test-negative case–control design. The study as reference 10 (Tartof et al., 2021) was conducted in population aged 12 and older in Southern California, United States, using retrospective design based on the healthcare system. In this review, the VE data from references 7 and 8 were for symptomatic COVID-19 disease, while the data from reference 9 and 10 were for all monitored SARS-CoV-2 infections.

Several vaccines have shown good efficacy in their phase 3 clinical trials, but only a few have completed relatively long-term follow-up for protection. As shown in Figure 1, the recent studies (Andrews et al., 2021; Chemaitelly et al., 2021; Lin et al., 2021; Tartof et al., 2021) of long-term VE for BNT162b2 (Figure 1A), mRNA-1273 (Figure 1B), AZD1222 (Figure 1C), and Ad26.COV2.S (Figure 1D) suggested that these vaccines differ widely on the extents of waning VE over time in symptomatic COVID-19 cases or asymptomatic infections caused by the Delta variant. As the world’s first COVID-19 vaccine was added to EUL, BNT162b2 has currently been approved for use in more than 100 countries, as well as the most studied vaccine. According to Figure 1A, in the context of the Delta variant as the dominant epidemic strain, the VE of BNT162b2 against symptomatic COVID-19 or infection overall appeared to peak 1–2 months after two doses (83.3–94.3%) and began to decrease apparently after 3 months. Waning VE was most pronounced in a one test-negative case–control study conducted in a population aged 12 and older in Qatar (Chemaitelly et al., 2021); the results showed that the VE of BNT162b2 against SARS-CoV-2 infection was less than 50% within 10–14 weeks after two doses and could not even be observed after 20 weeks—there was no difference in the incidence of infection between vaccinated and unvaccinated population. Lin et al. (2021) recently published a retrospective study that simultaneously evaluated the long-term VE against the symptomatic COVID-19 disease of BNT162b2, mRNA-1273, and Ad26.COV2.S in a population aged 12 and older in North Carolina, United States. In that study, the VE for BNT162b2 and mRNA-1273 against symptomatic disease within 1–3 months after two doses of vaccination peaked at 94.3 and 96.0%, respectively. However, the VE for BNT162b2 has decreased to 63.2% at 8–9 months, while mRNA-1273 remained more than 80% (Figures 1A,B). Since Ad26.COV2.S was not deployed until March 2021 in the United States and was suspended for a short period due to safety concerns, the information about its effectiveness is limited. Based on the available data in North Carolina, the effectiveness of a single dose of Ad26.COV2.S ramped to a peak level that seemed to be lower than the effectiveness of the two doses of mRNA vaccines 1 month after vaccination in the pre-Delta period, but there was little loss of VE caused by the Delta variant at 6–7 months (Figure 1D). In addition, a study from the United Kingdom (Andrews et al., 2021), using a test-negative design, demonstrated waning VE against symptomatic disease for BNT162b2 and AZD1222. Among individuals aged 16 and older with full vaccination, after 20 weeks, the VE for AZD1222 and BNT162b2 were both approximately 20% lower than their peaks (Figures 1A,C).

Furthermore, there is no significant correlation between the extent of waning VE and age, notwithstanding the lower peak VE in the elderly (Andrews et al., 2021; Chemaitelly et al., 2021; Tartof et al., 2021). Despite the fact that male sex has been found to experience more severe COVID-19 outcomes than females (Kelada et al., 2020), to our knowledge, there are no data to support the effect of sex on the level and waning of VE (Bruxvoort et al., 2021; Lin et al., 2021; Thomas et al., 2021). One good news is that there is also no evidence of the waning VE against hospitalizations and deaths. About half a year after the full vaccination, the VE against severe outcomes remains more than 75% for BNT162b2, mRNA-1273, and AZD1222 and more than 68% for Ad26.COV2.S (Bajema et al., 2021; Nunes et al., 2021; Self et al., 2021; Thompson et al., 2021). However, it is still too early to tell with the insufficient follow-up whether the waning VE against severe outcomes might occur after more time has elapsed, and it is unclear whether the Omicron variant will weaken VE further or faster.

Overall, based on the findings thus far, although the results vary from study to study, it is generally considered that VE might begin to decline over time around 3 months after the full vaccination. Among the licensed COVID-19 vaccines continuously monitored, the duration of VE against symptomatic infection for mRNA-1273 may be relatively optimistic, while almost all showed persistence in protecting against hospitalization and death after 6 months.

## What Factors Influence the Duration of Protection?

There are several contributing factors in the duration of VE, which might be summarized into three categories: research methodologies, vaccine-induced immune responses, and SARS-CoV-2 variants.

Different approaches to assess waning VE, including study design, objects, sites, and the statistical method, may yield different results. For instance, the statistical data from a randomized controlled trial, real-world test-negative case–control designed study, retrospective study attached to the public health system, and a lot more may have varying degrees of reliability and bias. It is important to note that the disproportionate risk of infection in individuals vaccinated early due to some strategy, the great diversity in means of obtaining confirmed cases or government interventions, and the changing dominant variants over time make the conclusions of studies conducted in specific settings need to be carefully employed. Although the data included in Figure 1 in this review were only for the Delta variant, as well as from very similar study periods and populations, there is a potential limitation that the results of different studies may not be comparable, with the confounding factors mentioned above. Nevertheless, longer-term follow-up studies with larger population sizes will provide a more consistent picture of waning VE, which is always critical for public health response and policy decisions.

The VE is undoubtedly closely related to the type and characteristics of the vaccine since the action mechanism of vaccines developed along different technical routes is different, such as the level of antibodies or the number of long-lived immune cells the vaccine induces. Yet, whether a neutralizing antibody (NAb) is necessary mechanism in mediating protection remains controversial. One study published in May 2021 (Khoury et al., 2021) suggested that the neutralization level was highly predictive of immune protection and predicted that the immune protection from infection may wane over time as neutralization levels decline based on a model. This prediction seems to be matched by subsequent studies on the durability of the antibody induced by mRNA vaccines (Andrews et al., 2021; Falsey et al., 2021). Collier et al. (2021) conducted a study on the differential kinetics of immune responses elicited by two mRNA vaccines (two doses) and Ad26.COV2.S (one dose) over an 8-month follow-up period. The results of the kinetics of live virus-neutralizing antibody responses showed that BNT162b2 and mRNA-1273 vaccines were characterized by high peak neutralizing antibody responses that declined sharply by 6 months (BNT162b2: 1,789–543; mRNA-1273: 5,848–1,524) and the responses declined further by 8 months (BNT162b2: 543–53; mRNA-1273: 1524–133), while the Ad26.COV2.S induced lower initial antibody responses but were relatively stable with minimal-to-no evidence of decline. However, with all three vaccines, there were generally stable antibody-dependent cellular phagocytosis and complement deposition responses, and there was no clear trend overall in the percentage of CD4 + and CD8 + T cells at 8 months. The data from Lin et al. (2021)’s study on the waning VE in North Carolina mentioned above seems to be perfectly explained by these antibody results, although the correlations of protection from SARS-CoV-2 are not yet defined. Conversely, some researchers believe that decreased neutralizing activity *in vitro* does not, on its own, predict that vaccines will be ineffective. Despite the fact that a decline was observed in neutralization response, vaccinees retained a neutralization capability against the Beta variant, possibly due to vaccination stimulating a cross-variant immune response (Corbett et al., 2021; Dejnirattisai et al., 2021; Stamatatos et al., 2021), as emerging evidence suggests that NAbs might not be the only mechanism of protection. T cells and non-NAbs are considered to also play a vital role in regard to the protection against COVID-19 (Geers et al., 2021; Reynolds et al., 2021; Tan et al., 2021; Tarke et al., 2021). SARS-CoV-2 spike-specific T-cell response induced upon COVID-19 vaccination or infection appears to be more robust and persistent than expected (Sekine et al., 2020; Collier et al., 2021), and remains responsive to the variants, including Omicron (Keeton et al., 2021), but the extent to which long-lived specific T cells contribute to protection needs to be studied in depth. Moreover, it turned out that a longer interval between the first and second doses of COVID-19 vaccines might confer stronger immune responses against SARS-CoV-2 (Payne et al., 2021; Voysey et al., 2021). It has been argued that the long dosing interval gives rise to T cells that are more typical of helper T cells and long-term memory T cells that promote the memory and generation of antibodies (Mahase, 2021). However, the concern with longer regimen is the increased infection risk during the interval when the single dose of most COVID-19 vaccines provides limited protection.

Indeed, the unknown aspect of the virus is the worst enemy in the fight against the future outbreak. The combination of the replication and recombination of the RNA of the virus in a huge number of individuals is conducive to the emergence of variants with improved transmissibility and increased immunological escape (Burkert et al., 2021; Krause et al., 2021), some of which are even untraceable. It is vital to remain vigilant and monitor the variants arising around the globe, as well as the trends during the outbreak.

On the basis of the available data, the mainstream viewpoint seems to be that the Delta variant had little effect on VE. In October, 2021, a published study (Lucas et al., 2021) on the effectiveness of the two mRNA vaccines against 16 epidemic variants revealed that Delta was less concerning for Nab escape than Gamma and Beta. Another study also found no significant difference in the extents of waning VE among different variants (Tartof et al., 2021). There have been data supporting that the rise in vaccine breakthroughs associated with Delta is more probably associated with its high transmissibility and increased viral load prior to symptoms (Bolze et al., 2021; Challen et al., 2021; Lopez Bernal et al., 2021; Lustig et al., 2021). Blaiszik et al. (2021) concluded in one study that the waning VE appears to be driven by the demographic factors affecting the composition of vaccinated cohorts, particularly as pertains to age distribution. Although the Delta variant currently had a negligible effect on VE, a continued adaptation of SARS-CoV-2 to human transmission and immune escape seems inevitable. The Omicron variant is believed to pose a grave threat to humans because there are at least 32 amino acid mutations that occurred in spike protein, which is the target antigen for current COVID-19 vaccines, while the number of that for Delta is only 15 (Jhun et al., 2021). The preliminary evidence suggested an increased risk of reinfection with this variant, as compared to other VOCs. However, with a limited knowledge of Omicron, data are needed to support whether existing vaccines can still protect against this variant.

## Future Considerations

Given the above, the factors influencing the duration of protection are intricate. Whether the wanning VE is the cause of the periodic outbreaks in waves remains a mystery. However, what is clear is that much more needs to be done to fill in the information gaps in COVID-19. On the one hand, a more indestructible public health system should be established to keep pace with SARS-CoV-2 variants and make decisions quickly based on strong clinical evidence. On the other hand, the rapid global deployment of licensed and effective vaccines remains an urgent and vital public health priority, while safer, more effective and broad-spectrum vaccines with different delivery routes still imminently need to be developed worldwide.

As part of effort, several studies on sequential booster vaccination have been carried out. Based on current results, the safety is reassuring; the immunogenicity and even the VE can be greatly improved after both homologous and heterologous booster vaccinations (Barda et al., 2021; Borobia et al., 2021; Choi et al., 2021; Falsey et al., 2021; Grange et al., 2021; Hall et al., 2021). By 1 month after dose 3 of BNT162b2 (administered at 7–9 months after the primary two-dose series), neutralization geometric mean titers (GMTs) against wild-type virus increased to more than five times as high (in 18-to-55-year-olds: 387–2,119) and to more than seven times as high (in 65-to-85-year-olds: 261–2,032) as the GMTs 1 month after dose 2, and a similar pattern was seen in the assays of neutralization GMTs against the Delta variant (Falsey et al., 2021). Meanwhile, BNT162b2 given as a second dose in individuals prime-vaccinated with a single dose of AZD1222 (the interval was 8–12 weeks) induced a robust immune response, with significantly higher GMTs after 14 days in the booster dose recipients than those who did not receive the booster dose (1,905.69 vs. 41.81; *P* < 0.0001) (Borobia et al., 2021). Moreover, a large-scale observational study (Barda et al., 2021) in Israel encouragingly demonstrated that the effectiveness of the third BNT162b2 dose administered at more than 5 months after the second dose, compared with two doses only, was estimated to be 93% against hospitalization, 92% against severe disease, and 81% against COVID-19-related death. The data from Moderna were also impressive; wild-type virus neutralization was 3.8-fold higher 2 weeks after 50 μg booster doses of mRNA-1273 (mean boost interval of 6.7 months), compared to peak titers measured 1 month after the primary series in healthy adults (Choi et al., 2021). It also emerged that the mRNA-1273 booster increased neutralization against the other VOCs or VOIs to the levels that were statistically equivalent to the wild-type benchmarks. It is interesting to note that Atmar et al. (2021) recently conducted a study on booster doses with nine different combinations based on three vaccines (BNT162b2, mRNA-1273, and Ad26.COV2.S), which reported that homologous boost increased neutralizing antibody titers 4.2–20-fold whereas heterologous boost increased 6.2–76-fold (mean boost interval of 13.7–24.1 months). There were signs that heterologous prime boost strategies might offer immunological advantages to optimize the breadth and longevity of protection achieved with currently available vaccines. However, little evidence has been published on the long-term VE of either homologous or heterologous booster regimen, and more data are needed to further support the VE of a booster dose against the newer variant. So far, more than 50 countries have confirmed COVID-19 vaccine booster or additional doses, although the WHO has not yet recommended booster doses due to the unbalanced allocation of vaccine resources (World Health Organization (WHO), 2021b). In addition, the relationship between measured immunity and clinical protection conferred after vaccination or natural infection conduces to plan the next steps in vaccine campaign. In most instances for now, it seems inappropriate to perform direct comparisons of neutralizing antibody titers among diverse human trials for different vaccines measured by different neutralization assays (Khoury et al., 2020). Although the WHO international standard (IS) for anti-SARS-CoV-2 immunoglobulin (NIBSC code 20/136) was available in December 2020, the standardization and harmonization in laboratory testing take some time to move on (Infantino et al., 2021). Another significant refinement should be to figure out the correlation between serological markers and protection against infection, disease or adverse outcomes, and to identify if any other markers provide a better predictive value than neutralization.

As natural immunity builds in the population, SARS-CoV-2 variants may be increasingly selected as immune escape variants. It is almost unlikely that a booster shot of vaccine based on the original strain of SARS-CoV-2 would always be able to block out the variants. The reestablishment of antigenic composition is in urgent need to fight against the emergent VOCs. According to the official statement, Pfizer and BioNTech have already started the development of a variant-specific vaccine for Omicron and expected to have it available by March 2022. It should be also noted that effective vaccination strategies need not be restricted to a single route. There are eight intranasal spray COVID-19 vaccines currently under clinical development worldwide according to the WHO (World Health Organization (WHO), 2021a); however, no intranasal COVID-19 vaccine has been approved for use to date. Compared to intramuscular vaccines, intranasal vaccines provide two additional layers of protection: one is vaccine-induced IgA antibody, and the other is resident memory B and T cells in the respiratory mucosa, which are considered as powerful weapons in the prevention of respiratory infections in the whole population. One study conducted by Shin and Iwasaki (2012) indicated that memory cells primed by intramuscular vaccination can be “pulled” into mucosal sites by subsequent mucosal vaccination. When one plus one is greater than two, the ideal vaccination strategy might use an intramuscular vaccine to induce a long-lived systemic humoral response and a broad repertoire of central memory B and T cells, followed by an intranasal booster that recruits memory B and T cells to the nasal passages and further guides their differentiation toward mucosal protection, including IgA secretion and tissue-resident memory cells in the respiratory tract (Lapuente et al., 2021; Lund and Randall, 2021). Ultimately, the goal of vaccination is to elicit long-lived protective immunity, and in addition to developing new vaccines and increasing coverage, innovative strategies might be a shortcut to achieve this.

## Conclusion

The benefits of vaccination in reducing the risk of COVID-19 disease are clearly supported based on current evidence. Sustained and protective immunity in the population is the key to end the pandemic, which is exactly what the countries around the world need to make efforts to achieve in the years ahead. However, a continued adaptation of SARS-CoV-2 to transmission and immune escape appears to be inevitable. Facing the complex and uncertain situation, it might be urgent and effective to make continued efforts to optimize vaccine, implement prime and booster campaigns, and further explore multiple and additional layers of protection against infection. It is also highly important to monitor the evolution and mutation patterns of SARS-CoV-2, which make it possible to establish a forecasting model for viral mutations. The knowledge on the characteristics of future variants may enable shortened timelines to vaccine and therapeutic drug development and help in the control of future COVID-19 outbreak.

## Author Contributions

CZ, XL, QC, and YuS searched the information and collected the data. CZ drafted the manuscript. TW, YiS, SH, and NX revised the manuscript. All authors provided critical feedback and contributed to the final version of the manuscript.

## Conflict of Interest

The authors declare that the research was conducted in the absence of any commercial or financial relationships that could be construed as a potential conflict of interest.

## Publisher’s Note

All claims expressed in this article are solely those of the authors and do not necessarily represent those of their affiliated organizations, or those of the publisher, the editors and the reviewers. Any product that may be evaluated in this article, or claim that may be made by its manufacturer, is not guaranteed or endorsed by the publisher.
